# Low temperature tolerance of three *Aedes albopictus* strains (Diptera: Culicidae) under constant and fluctuating temperature scenarios

**DOI:** 10.1186/s13071-020-04386-7

**Published:** 2020-11-23

**Authors:** Lisa Tippelt, Doreen Werner, Helge Kampen

**Affiliations:** 1 Friedrich-Loeffler-Institut, Federal Research Institute for Animal Health, Greifswald, Insel Riems Germany; 2 Leibniz Centre for Agricultural Landscape Research, Muencheberg, Germany

**Keywords:** Asian tiger mosquito, Cold acclimation, Cold hardiness, Constant temperatures, Diapause, Fluctuating temperatures, Hatching, Overwintering, Temperature tolerance

## Abstract

**Background:**

*Aedes albopictus*, a vector of numerous viruses and filarial worms, has already established in 20 countries in Europe, mainly colonising subtropical regions. Continuing adaptation to climatic conditions in temperate areas would probably result in a spread to more northern European countries, producing an increasing risk of mosquito-borne pathogen transmission over a much greater area. Based on previous studies showing that *Ae. albopictus* is able to overwinter in Germany, this study aims to determine more exactly its ecological limits of enduring low temperatures.

**Methods:**

Non-diapausing and experimentally induced diapausing eggs of three different *Ae. albopictus* strains (tropical, subtropical and temperate origins) were exposed to four different regimes with constant temperatures and three different regimes with fluctuating temperatures in a course of a day for a minimum of 2 and a maximum of 30 days. The hatching rate of larvae after cold exposure of the eggs was taken as a measure of cold tolerance.

**Results:**

The experiments showed that the tropical *Ae. albopictus* strain had a lower cold tolerance than the subtropical and the temperate strains. The eggs of all used strains were able to survive constant temperatures as low as −5 °C for an exposure period of 30 days, while constant temperatures as low as −10 °C were endured for 2 days by the tropical strain and for 10 and 20 days by the subtropical and temperate strains, respectively. At fluctuating temperatures, both the subtropical and the temperate strains exhibited hatching under all temperature regimes, even with a minimum temperature of −10 °C, whereas the tropical strain ceased hatching after an exposure period of 30 days under the temperature regime with a minimum temperature of −10 °C. The analyses showed that the temperature played the major role in interpreting the hatching rates of the eggs. The condition, whether the eggs were diapausing or not, had no significant influence, although results indicated a slightly higher cold tolerance of diapausing eggs at −10 °C.

**Conclusions:**

It must be expected that subtropical and temperate strains of *Ae. albopictus* are able to withstand common central European winters and are able to establish in considerable parts of the continent.
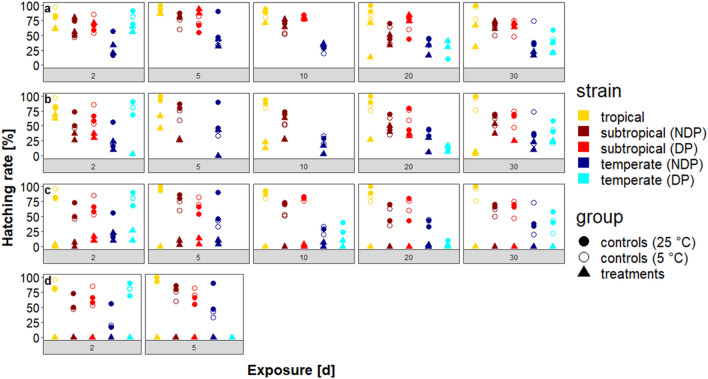

## Background

The Asian tiger mosquito, *Aedes albopictus* (Skuse, 1894), is a thermophilic invasive mosquito species originating from tropical and temperate regions in southeastern Asia [1]. It is active during the day and feeds on both animals and humans, with a preference for human hosts [2]. The species is a feared vector as it is able to transmit more than 20 viruses and filarial worms pathogenic to animals and humans [3–5]. Since the beginning of this millennium, cases of autochthonous mosquito-borne diseases like chikungunya and dengue have substantially increased in southern Europe (e.g. in Italy, France and Croatia), probably mediated by established *Ae. albopictus* populations [6–11]. In 2018, the first autochthonous cases of dengue occurred in Spain [12] which is not surprising as infected *Ae. albopictus* had been collected in the field already in 2015 [13].

Within the last 30 years, *Ae. albopictus* has managed to spread from its native distribution area to all continents except Antarctica [5]. The trade in used tyres and ornamental plants such as ‘lucky bamboo’ (*Dracaena* spp.) has played an important role in its global dispersal [14]. In Europe, the species was first detected in 1979 in Albania [15], but only a further introduction event to Genoa, Italy, in 1990 led to its rapid spread across southern Europe, probably facilitated by favourable climatic conditions [16]. At present, *Ae. albopictus* has been found in 28 European countries and is established in 20 of them [17, 18]. The recent detections in Germany, England and Portugal demonstrate its continuing spread throughout Europe [19–21].

The first detection of *Ae. albopictus* in Germany occurred in 2007 in the form of eggs [22], followed by findings of adults in 2011 and 2012 [23–25]. For several years, all females caught in Germany were assumed to have been introduced *via* motorways from southern Europe [22, 24], and local reproduction was regarded highly unlikely due to unsuitable climatic conditions. Only in 2014, larvae and pupae were collected in southern Germany over several months, demonstrating reproductive potential [26]. Repeated overwintering of *Ae. albopictus* has later been demonstrated in Germany, and several populations are now considered established [27–29]. Modelling approaches based on climatic parameters suggest that a spread to other regions of Germany is possible [30–33].

The ability of *Ae. albopictus* to survive winter temperatures is crucial for its establishment in temperate regions [34]. *Aedes albopictus* is known to overwinter as a pharate larva in the egg stage *via* diapausing [35]. Diapause is a form of dormancy linked to a strongly reduced metabolic activity as a result of seasonal adaptation, although diapause is not strictly linked to the winter season. In contrast to quiescence, diapause is not reversible by flooding as a hatching stimulus once initiated [36]. Pharate larvae will only get susceptible to this stimulus as soon as diapause is terminated by an increase in temperature and a prolongation of the daily photoperiod [37]. Only *Ae. albopictus* from subtropical and temperate regions are able to overwinter by producing diapausing eggs while tropical strains reproduce continuously during all seasons [38]. Naturally, diapause is induced in the pupa or adult of the female parent under autumnal conditions, characterized by a progressive decrease in photoperiod length and temperature [39]. Laboratory experiments demonstrated that the photoperiod plays a more decisive role than the temperature. In fact, temperatures as high as 25–27 °C together with a short photoperiod were able to induce a diapause response but this response almost or even totally diminished in mosquitoes reared at 29 °C [40]. A temperature of 21 °C has proven most reliable for inducing diapause [40].

It is well known that diapausing eggs have a higher tolerance against low temperatures than non-diapausing eggs [41, 42]. Also, both laboratory and field experiments have shown that tropical *Ae. albopictus* strains have a lower tolerance against low temperatures than temperate strains [42–44]. Furthermore, it could be demonstrated that strains originating from subtropical regions exhibit a tolerance against low temperatures that is intermediate between those of temperate and tropical strains exposed to low temperatures for 6 days [41]. Thomas et al. [42] examined the cold tolerance of a tropical and a subtropical strain and could demonstrate that non-diapausing eggs of the tropical strain withstand − 10 °C and diapausing eggs of the subtropical strain even survive exposure to − 12 °C for one hour. However, the authors only considered short-term exposure to constant low temperatures with a maximum exposure period of 24 h. Results indicate that hatching could be possible for exposure periods longer than 24 h at temperatures higher than −10 °C.

This study aimed at finding the limits of egg survivability after long-term cold exposure of up to 30 days under constant and fluctuating temperature scenarios. Thus, the intention of the study was not to produce high experimental hatching rates but to check whether hatching is possible at all and to elucidate ecological limits.

## Methods

### Origin and keeping of mosquitoes

Three *Ae. albopictus* laboratory strains were used: one originating from Mauritius, which will be called ‘tropical strain’ for the purpose of this paper; one from Rimini, Italy, called ‘subtropical strain’; and one from Freiburg, Germany, called ‘temperate strain’. The first two strains were long-maintained laboratory colonies with more than 70 generations, whereas the temperate strain was collected in the field in August 2015 and at the time of the study had developed 20 generations in the laboratory. All strains were reared according to a standard protocol also used by Thomas et al. [42] under constant temperature (25 ± 1 °C), relative humidity (RH) (70 ± 5%) and light regime (12 h light:12 h darkness). Experiments with tropical *Ae. albopictus* strains are commonly conducted at a temperature of 27 °C [45], whereas experiments with strains of different origins, including temperate ones, are mostly done at lower temperatures, such as 25 °C [39, 44]. In our experiments, we applied this lower rearing temperature to provide better conditions for the subtropical and temperate strain.

Larvae were reared in basins filled with stale tap water and fed with ground fish food (TetraMin; Tetra, Melle, Germany). Pupae were transferred to beakers filled with up to 250 ml of stale tap water, which were placed in cages (30 × 30 × 30 cm^3^; Watkins & Doncaster, Leominster, UK) for adult emergence. Adults were provided with a sugar solution (8% fructose) *ad libitum*. Once a week, females were membrane-fed with bovine EDTA-blood warmed to 37 °C *via* an artificial feeding system (Hemotek, Blackburn, UK). Beakers filled with stale tap water and equipped with standard filter paper that protruded from the water were offered for oviposition. After drying the filter papers with attached eggs in Petri dishes for 3 to 4 days, the dishes with the still moist filter papers were sealed with parafilm (Bemis Company, Neenah, Wisconsin, USA) to minimise further desiccation. Dishes were stored in the insectary until further processing.

For the experiments, non-diapausing eggs of the tropical, subtropical and temperate strain as well as diapausing eggs of the subtropical and temperate strains were used. Before starting the experiments, it was checked if laboratory conditions had affected the diapause response of the tropical strain. It could be confirmed that the tropical strain was still not susceptible to diapausing conditions. For producing diapausing eggs, larvae of the subtropical and the temperate strains not older than L3 stage were transferred in beakers to a climate chamber with a photoperiod of 8 h light:16 h darkness, a constant air temperature of 20 ± 1 °C and a relative humidity of 70 ± 5%. Procedures for larval feeding, blood-feeding, oviposition and egg storage were not changed.

### Preparation of eggs

To avoid age further influencing analyses as a covariate, the non-diapausing eggs used in this study had a similar age of about 3 (minimum 2.5, maximum 4) months. This is the age at which the diapause state is terminated, and eggs are susceptible to hatching stimuli [41]. Around 60 (56–69) eggs from the same oviposition cycle were split into two subsets of around 30 eggs transferred to separate wooden spatulas with the aid of a brush, leading to two treatments and controls each for the same strain and the same rearing conditions (routine laboratory rearing conditions and diapause-inducing conditions), temperature treatment and exposure period. Hence, two spatulas with *c.* 30 eggs each were exposed simultaneously to exactly the same conditions. By means of a stereomicroscope, it was verified that no eggs showing signs of desiccation or empty egg-shells produced by spontaneous hatching were used for the experiments. Each spatula was placed into a separate Petri dish sealed with adhesive tape. The Petri dishes were kept in an upright position in open plastic boxes, which were transferred into a climate chamber (KBWF 720; Binder, Tuttlingen, Germany; temperature variation: ± 0.5 °C) programmed to run the desired temperature regime.

As the diapausing populations did not reproduce as efficiently as the non-diapausing populations, diapausing eggs were of limited availability, especially in the temperate strain. Therefore, analysis of diapausing populations was not possible for all exposure periods, and treatments at higher temperatures had to be cut. By all means, at least the minimum and the maximum exposure periods of 2 and 30 days, respectively, were considered.

### Cold exposure

**Fig. 1:**
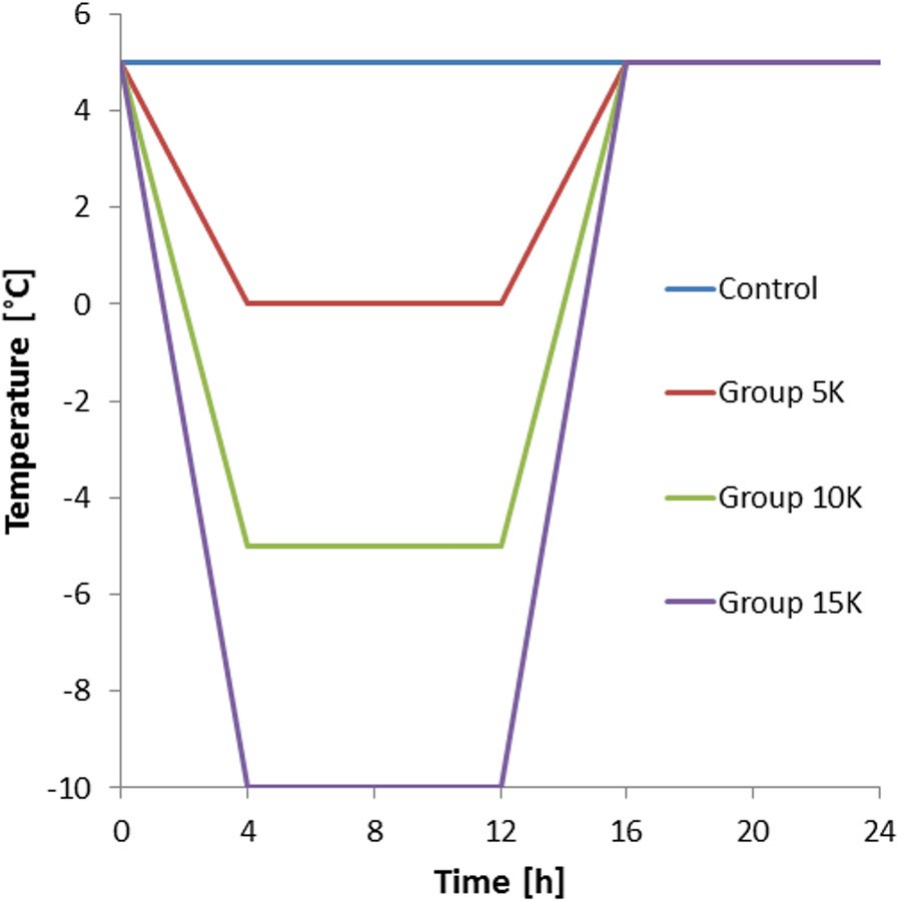
Daily temperature course of the three fluctuating temperature regimes

Two experimental setups were followed, one with exposure of eggs to constant temperatures and one with exposure of eggs to fluctuating temperatures. The first study was carried out using four different temperatures (0, − 5, −10 and −15 °C) and five different exposure periods at each of these temperatures (2, 5, 10, 20 and 30 days). In the second setup, three different cooling-warming cycles were applied, with a maximum temperature of 5 °C in all treatments and different minimum temperatures. The minimum temperatures were set to 0, −5 or −10 °C, respectively, leading to a temperature difference of 5, 10 and 15 K. Accordingly, these different treatments will be called ‘5K group’, ‘10K group’ and ‘15K group’ hereafter. All fluctuating temperature treatments underwent a standardised day-night temperature cycle with an exposure period of 8 h at the maximum temperature of 5 °C, an exposure period of 8 h at the minimum temperature (0, − 5 or −10 °C) and 4 h, each, for reaching the respective minimum and maximum temperatures (Fig. 1). For the experiments, it was important to choose a maximum temperature above, but close to, 0 °C because of two reasons. First, a maximum temperature slightly above the freezing point is common in many areas in Germany during day time in winter and was given in field studies with *Ae. albopictus* in northeastern and central Germany [29, 46]. Secondly, the chosen temperature threshold allows testing three different temperature amplitudes traversing the freezing point twice in every temperature cycle.

Eggs were exposed to these fluctuating temperature cycles for the same time periods as those exposed to constant temperatures, i.e. 2 to 30 days. For comparison, two sets of controls of the same populations were exposed to routine insectary temperature (25 ± 1 °C) or to a constant temperature of 5 °C, respectively. Temperature was measured in the insectary or the climate chamber, respectively. The first control was used as a comparison to optimal developmental summer conditions and is called ‘summer control’. By contrast, the second control, called ‘winter control’, was only applied for the fluctuating temperature scenario and should demonstrate the hatching rate for the maximum temperature of this temperature scenario. Hence, it serves as an internal control to test if the maximum temperature itself, without any fluctuations, could influence the hatching of the eggs. During all treatments (5 to −10 °C), eggs were kept in the dark.

### Procedure after cold treatment

After having passed the set temperature regimes, eggs were counted again and flooded by transferring the spatulas into beakers filled with 300 ml stale tap water, according to previous studies [47, 48]. The number of hatched larvae was recorded during the whole flooding process, with larvae found dead treated just as living ones since both obviously succeeded in hatching. After two weeks of flooding, the spatulas were removed from the beakers and dried for one week. By filtrating the water of each beaker with the aid of common filter paper, detached eggs were retained and re-attached onto the spatulas using a brush. After drying for one week, the spatulas were flooded for another week. Former experiments with the same strains had shown that this procedure will lead to hatching rates of 60–90% in the controls of the tropical and subtropical strains kept at 25 °C [46]. Hatching was taken as a measure for the eggs to survive treatment. Non-hatched eggs were not checked for larvae being viable or not and were considered dead. According to studies with other insects, low temperatures do not only affect mortality of eggs but of later life stages as well, denoted as delayed mortality [49, 50]. However, such a phenomenon did not play a crucial role in *Ae. albopictus* [41]. Therefore, this study only aimed for the egg stage, i.e. the hatching rates, and did not track the impact of low temperatures during the egg stage on later life stages.

### Statistical analysis

For statistical analysis, calculated hatching rates were compared. The influence of five different factors on the response variable, the hatching rate, was examined: (i) strain: three levels (tropical, subtropical and temperate strains); (ii) condition: two levels (no diapause and diapause); (iii) temperature: six levels (25 °C, 5 °C, 0 °C, −5 °C, −10 °C and −15 °C). For the fluctuating temperature scenario, the minimum temperature was used in the analyses. The factor ‘temperature’ is also suitable for differentiating between treatments and controls as only controls were exposed to 25 °C or 5 °C, respectively; (iv) exposure: nine levels (16 h, 40 h, 48 h (2 days), 80 h, 120 h (5 days), 160 h, 240 h (10 days), 480 h (20 days), 720 h (30 days)). For better comparing treatments with constant and fluctuating temperatures, the exposure periods at the minimum temperature were used for the analyses in the case of the fluctuating temperature scenario, whereas the whole exposure period was considered in the case of the constant temperature scenario; (v) scenario: two levels (constant and fluctuating)

Data were square root-transformed for reaching normality followed by multifactorial ANOVA. After conducting an ANOVA, a Tukey’s honestly significant different (HSD) *post-hoc* test was applied for identifying the decisive levels of the significant factors. For examining the hatching pattern, the hatching results were split into three categories, ‘normal hatching’ (without delay), ‘delayed hatching’ and ‘no hatching’, followed by calculating Cramér’s V for estimating the effect size of each factor. For all statistical tests as well as for the preparation of figures, the software R (version 3.5.2.) was employed, supported by the packages *lsr* [51], *ggplot2* [52], *ggpubr* [53] and *vcd* [54].

## Results

### Constant temperature scenario

**Fig. 2:**
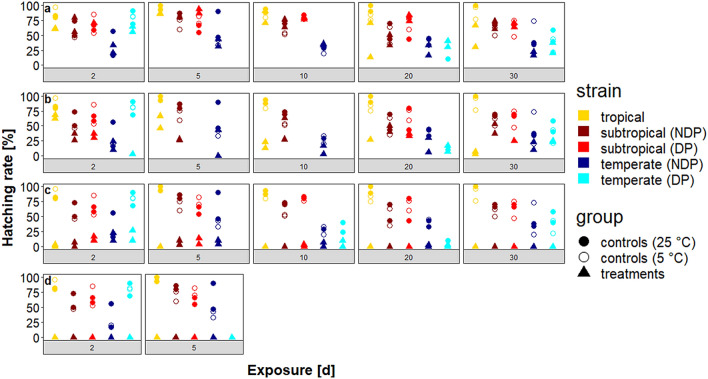
Hatching rates of the various *Ae. albopictus* strains after exposure to constant temperatures (NDP, no diapause; DP, diapause). **a** 0 °C. **b** −5 °C. **c** −10 °C. **d** −15 °C. Control eggs were kept at a constant temperature of 25 °C (filled circles) or 5 °C (blank circles), respectively

All three mosquito strains showed hatching after exposure to 0 °C and − 5 °C for up to 30 days (Fig. 2). The − 10 °C temperature approach revealed great differences between the strains (Fig. 2c) and produced hatching rates with a maximum of about 25% (2–20 days exposure, depending on the strain) and samples with no hatching at all (5–30 days exposure, depending on the strain). No hatching at all occurred after exposure to −15 °C for 2 and 5 days (Fig. 2d). Because of the latter, no treatments with longer exposure periods were tested.

The hatching rates of the controls of the tropical strain were generally very high and varied between 80–100%. After exposure to 0 °C, no significant decrease of the hatching rates was observed (all *P* > 0.05, Fig. 2a). By comparison, treatments showed a decrease in hatching rates after exposure to temperatures of − 5 and − 10 °C, and hatching finally ceased altogether after exposure to −10 °C for longer than 2 days (Fig. 2c). A significant decrease of the hatching rate was observed after exposure periods of 10 and 30 days at −5 °C (*P* < 0.01), but not after an exposure period of 20 days at the same temperature (*P* > 0.05, Fig. 2b).

The controls of the subtropical strain reached hatching rates ranging from 43–87%, which were generally lower than those of the tropical strain. Treatments exhibited no conspicuous decrease of hatching rates after exposure to constant temperatures of 0 and −5 °C in both non-diapausing and diapausing eggs (Fig. 2a, b). However, after exposure to −10 °C, hatching rates strongly decreased, and hatching ceased after exposure periods of 10 and 20 days, respectively (Fig. 2c). In this temperature regime, hatching rates of the non-diapausing eggs greatly varied from those of the diapausing ones, with the latter being able to endure the temperature twice as long (10 *vs* 5 days). After an exposure to −10 °C for 20 days, neither non-diapausing nor diapausing eggs showed hatching (Fig. 2c). As related to the controls, there was a significant decrease in hatching rates of non-diapausing eggs after an exposure to − 10 °C (all *P* < 0.001) and in the diapausing eggs after exposure periods longer than 2 days (all *P* < 0.05).

Of all examined strains, the controls of the temperate strain were the ones with the lowest hatching rates which rarely exceeded values of 50% in both non-diapausing and diapausing eggs. Only three controls of the diapausing eggs reached higher values (69 and 90% in the two-day-controls and 59% in one 30-day-control, respectively). Like the subtropical strain, treatments of the temperate strain showed no significant decrease of hatching rates as temperatures went down to −5 °C, albeit generally presenting much lower hatching rates (Fig. 2a, b). Hatching rates of the treatments of both non-diapausing and diapausing eggs of the temperate strain also generally remained below 50%, but some exceptions occurred in the diapausing eggs (Fig. 2a). At −10 °C, hatching of non-diapausing and diapausing eggs still occurred after 20 days representing the longest exposure period with hatching of all tested strains and conditions (non-diapausing and diapausing eggs). There were significant decreases in hatching rates of the non-diapausing eggs after exposure periods of 5, 20 and 30 days (all *P* < 0.01) but of the diapausing eggs only after an exposure period of 30 days (*P* < 0.01).

### Fluctuating temperature scenario

**Fig. 3:**
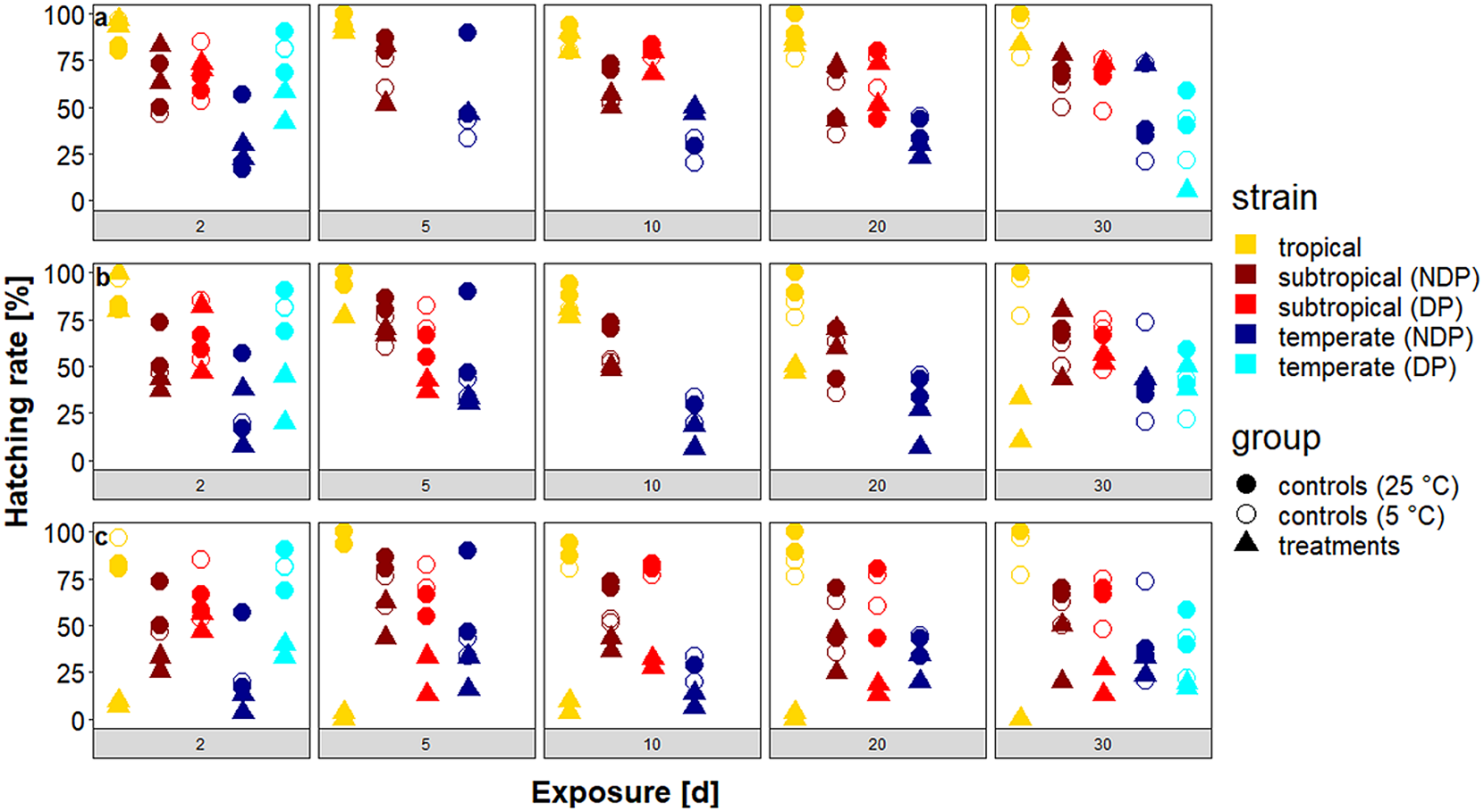
Hatching rates of the various *Ae. albopictus* strains after exposure to fluctuating temperatures (NDP, no diapause; DP, diapause). **a** 5 to 0 °C (5K group). **b** 5 to  5 °C (10K group). **c** 5 to  10 °C (15K group). Control eggs were kept at a constant temperature of 25 °C (filled circles) or 5 °C (blank circles), respectively

Under fluctuating temperatures, hatching rates of the controls were generally slightly lower than those obtained under the constant temperature scenario (when referring to controls in the fluctuating temperature scenario, summer controls are meant). The tropical strain had hatching rates between 47–100% in the controls, whereas the treatments exhibited hatching in almost all temperature regimes with a conspicuous decrease in hatching in the 10K group (Fig. 3a, b).

Hatching rates in the 15K group did not exceed values of 10% after all exposure periods and had a complete shortfall of hatching after an exposure period of 30 days (all *P* < 0.001, Fig. 3c). Hence, the treatments exposed for 20 days were the last ones with hatching. Similar to the treatments at constant temperatures, the ones at fluctuating temperatures showed no significant decrease in hatching rates in the 5K group, while in the 10K group distinct decreases in hatching were observable after exposure periods of 20 and 30 days, with significance in the latter (*P* < 0.01, Fig. 3b).

The hatching rates of the controls of the subtropical strains varied between 47–85% in both non-diapausing and diapausing eggs. Treatments showed hatching in all three groups for both physiological egg conditions, without any significant decreases at minimum temperatures of 0, −5 and −10 °C (Fig. 3). This was also the case for the maximum exposure period of 30 days (Fig. 3c).

As in the constant temperature scenario, the controls of the temperate strain had the lowest hatching rates of all controls, mostly exhibiting values below 50%, but in two cases above 80%. Treatments of this strain showed hatching in all groups and after all exposure periods without any significant decrease in hatching rates (Fig. 3).

### Influencing factors

The statistical analysis demonstrated that the factor ‘temperature’ had the greatest effect on the hatching rate (*F*_(5, 368)_  = 164.86, *P* < 0.001), followed by the factors ‘strain’ (*F*_(2, 368)_  = 40.08, *P* < 0.001), ‘scenario’ (*F*_(1, 368)_  = 19.41, *P* < 0.001), ‘exposure’ (*F*_(8, 368)_  = 10.48, *P* < 0.001) and ‘condition’ (*F*_(1, 368)_  = 2.26, *P* = 0.134). Hence, all examined factors except ‘condition’ had a significant influence on the hatching rate. The Tukey’s HSD *post-hoc* test could specify that the significance of the factor ‘strain’ was related to the temperate strain. Furthermore, there were significant interactions between the temperatures 25 °C and −5, −10 and −15 °C (all *P* < 0.001), leading to a significant, probably cold-related, decrease in the hatching rates.

### Hatching pattern

**Fig. 4:**
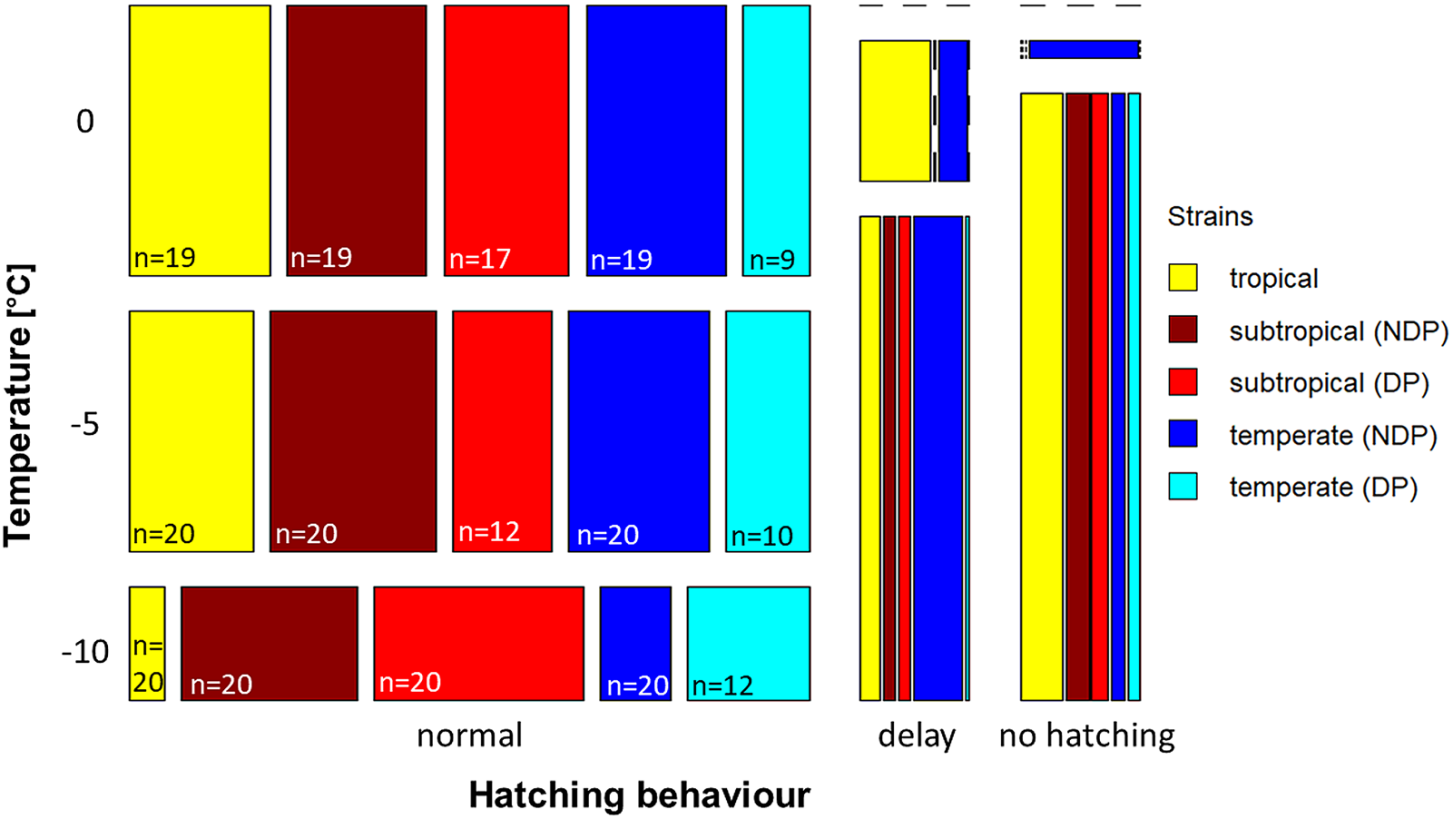
Overview about the hatching patterns in relation to the temperature and the used strains (NDP, no diapause; DP, diapause). The width of the rectangles corresponds to the relative proportion of the particular hatching pattern at the same temperature and the length of the rectangles to the proportion of the particular hatching pattern within the same strain. Horizontal dashed lines indicate the absence of the respective pattern. Vertical lines marked in the colour of a certain strain indicate the absence of the respective hatching pattern in this strain. The term ‘normal’ stands for hatching without delay, ‘n’ indicates the number of treatments per strain at the different temperatures

The eggs in the experiments were flooded for a total period of three weeks with an intermediate break of one week. All strains showed a hatching peak at the beginning of both flooding periods. However, in the treatments exposed to constant temperatures of −5 and −10 °C, strikingly many treatments exhibited hatching only during the second flooding period but not during the first. A mosaic plot depicts the dependency of the temperature on the hatching pattern for all treatments at the different temperatures (Fig. 4).

Hatching in both flooding periods (normal hatching) predominated after exposure to temperatures of 0 and −5 °C. Delayed and no hatching did not play any role after exposure to 0 °C. However, delayed hatching occurred more frequently after exposure to −5 and, particularly, −10 °C in comparison to 0 °C. After exposure to a temperature of −5 °C, the tropical strain and non-diapausing eggs of the temperate strain showed hatching only in the second flooding period (delayed hatching), whereas after exposure to −10 °C, treatments of diapausing and non-diapausing eggs of all strains displayed delayed hatching, most frequently in the previously mentioned strains. This translates to the conclusion that right before the complete lack of hatching, i.e. after the preceding exposure period, delayed hatching or single shortfalls were present in all strains. After exposure to −15 °C, exclusively ‘no hatching’ results occurred in all strains (data not shown).

Statistical analyses resulted in highly significant *P*-values for each factor (*P* < 0.05). The calculation of Cramér’s V produced the largest value for ‘temperature’ (CV = 0.60), followed by ‘scenario’ (CV = 0.22). The other factors had CVs between 0.19–0.12.

## Discussion

The data obtained in this study clearly demonstrate that the ability of hatching after cold exposure and, thus, the tolerance against low temperatures is dependent on the origin of the strain, the specific exposure temperature and the duration of exposure to minimum temperatures. In the present study, it could be shown that both minimum temperature and exposure period to minimum temperatures play significant roles for the outcome of the hatching results. The importance of temperature was also stressed in other experiments [42, 43, 55]. In agreement with the findings of Thomas et al. [42], our results further suggest that the exposure period becomes highly important at thermal limits of survival, i.e. at −10 °C.

The statistical analysis of the hatching rates of the different *Ae. albopictus* strains used in this study also indicate that their origins have an impact on their cold hardiness. Despite the limited sample size, the results allow a precise differentiation between the tropical *Ae. albopictus* strain on the one side, and the subtropical and the temperate strains on the other side due to the remarkably high capability of resisting low temperatures in the latter two. This finding is in agreement with other laboratory and field studies with North American, Asian and European *Ae. albopictus* strains [42–44, 46, 55, 56].

Interestingly, the subtropical and tropical strains did not differ significantly regarding hatching rates, but their hatching rates were quite different from those of the temperate strain. However, if the longest exposure periods with hatching are considered, the subtropical and temperate strains were more similar and differed tremendously from the tropical strain, which becomes most obvious by the hatching results at a constant temperature of −10 °C. The statistical significance between the temperate strain on the one side and the tropical and subtropical strains on the other might be caused by the overall low hatching rates in both controls and treatments of the temperate strain. This phenomenon also occurred in previous field experiments with the same strains and was thought to be attributed to incomplete adaptation to laboratory conditions [46]. Consequently, a field *vs* laboratory effect cannot be ruled out and can only be verified or falsified by testing the cold tolerance of the temperate strain again when adapted to laboratory conditions for a much longer time. In spite of this, the temperate strain was the only strain exhibiting hatching after an exposure period of 20 days at −10 °C. Thus, the temperate strain appears to be somewhat more cold-hardy, possibly caused by having gone through a bottleneck during its first winters in Germany, as has been described for populations in North America [57]. It is assumed that the German *Ae. albopictus* population (temperate strain) originates from Italy [24, 25], the same country, where the subtropical strain comes from. Genetic analyses in fact suggest that the population of Freiburg is closely related to one from northern Italy [27].

In addition to laboratory effects, several other factors, not considered in the present study, might influence the cold hardiness of *Ae. albopictus* such as maternal effects [39], genetic drift [58] or rapid adaptation [59], which occurred during adaptation to conditions in Germany. Hence, these factors also have to be taken into account when interpreting the different cold hardiness of the temperate and subtropical strains.

The tropical and subtropical strains used in this study were the same as examined by Thomas et al. [42], allowing direct comparisons of the hatching results. Based on these, tolerance of the tropical strain against low temperatures is higher in the present study. Thomas et al. [42] demonstrated that eggs exposed to a constant temperature of −10 °C for longer than one hour did not produce larvae. By contrast, the experiments conducted in this study still demonstrated hatching after exposure periods of 30 days at −5 °C and of 2 days at −10 °C. For the subtropical strain, Thomas et al. [42] showed endurance for four hours at −10 °C, as compared to 5 days for non-diapausing eggs and 10 days for diapausing eggs of the same strain in this study. One main reason for these divergent results could be physiological adaptation to the long rearing in the laboratory. Both strains used in the present study have passed more generations in the laboratory and therefore have a longer rearing history than the ones used by Thomas et al. [42]. A well-known problem in rearing insects and their adaptation to laboratory conditions is the genetic selection due to artificial and isolated keeping [60]. However, the hatching results obtained after low temperature exposure in both the tropical and subtropical strains demonstrated that rearing conditions apparently had not substantially reduced the capability of the laboratory strains to withstand low temperatures.

In a former field study using the same tropical, subtropical and temperate strains, eggs were able to hatch after short-term exposure to a minimum temperature of −6 °C. Furthermore, eggs of the subtropical strain were able to hatch after an exposure period of two hours at −10 °C [46]. Additionally, field data demonstrated that an *Ae. albopictus* strain from Jena in central Germany can withstand −10 to −11 °C for four hours [29]. The results of the laboratory experiments carried out in this study are in agreement with those findings as laboratory experiments showed that hatching also occurred after exposure to −10 °C. In contrast to the previously discussed factors, the factor ‘condition’, i.e. using non-diapausing or diapausing eggs, did not significantly influence the hatching rates in the experiments. These findings fit very well to those produced by Hanson & Craig [41] who described diapause as having a lower effect on the tolerance against low temperatures in *Ae. albopictus* than cold acclimation. However, if the thermal limits are considered, especially the treatments of eggs exposed to a constant temperature of −10 °C, diapausing eggs seemed to have an advantage, although this could be demonstrated for the subtropical strain only (Fig. 2c). The experiments conducted by Thomas et al. [42] provided an equivalent indication, thus supporting the findings of our study. Although short-term exposure (up to four hours) of non-diapausing eggs could endure −12 °C for a longer time than the diapausing ones in that study, diapausing eggs were able to withstand −10 °C for a longer exposure period than their non-diapausing counterparts. As in our study, those experiments were not able to find a significant difference between survival temperatures of the different ‘conditions’.

As diapause is a complex process mainly influenced by a gradual decrease of photoperiod and temperature, it is questionable if larvae and pupae held under constant conditions of low photoperiod and temperature instead of a gradual decrease will give rise to females that behave in a similar way as their natural populations.

In the experiments applying a fluctuating temperature scenario, the hatching rates of the treatments were higher than under a constant temperature scenario. This is quite logical, as the net exposure time to sub-zero temperatures under a fluctuating temperature scenario was only one-third of that of the exposure time to the same temperature under a constant temperature scenario. However, this difference is also present when comparing the absolute exposure time to minimum temperatures. This could be shown best for the hatching rates of the tropical and subtropical strains under a constant temperature of −10 °C as compared to a fluctuating temperature regime with minimum temperatures of −10 °C. At a constant temperature of −10 °C, no hatching occurred after exposure periods of 5 days and more in the tropical strain. However, under a fluctuating temperature regime with a minimum temperature of −10 °C (15K group) hatching even occurred after an exposure period of 20 days, which corresponds to a total net exposure time of about 5 days at −10 °C. A possible reason for this could be that cold acclimation occurred. All eggs used for the treatments had not been cold-acclimated prior to the experiments, but treatments exposed to a fluctuating temperature scenario had the chance of acclimation to low temperatures during the experiment as they experienced a daily period of 5 °C for eight hours. As mentioned before, Hanson & Craig [41] demonstrated a significant influence of cold acclimation on the tolerance of *Ae. albopictus* against low temperatures by keeping eggs for 60 days at 10 °C prior to the cold experiments. The authors conceded that an optimal temperature for cold acclimation could not be found. Therefore, it is not clear how long a duration at 5 °C would be necessary to cause such an acclimation effect.

Interestingly, both the eggs of the subtropical and of the tropical strains seemed to have undergone some kind of cold acclimation. That came unexpected in the case of the tropical strain, as cold acclimation could not be shown in other studies using tropical non-diapausing *Ae. albopictus* strains [41, 61]. However, in a field study with the same tropical mosquito strain as in this study, cold acclimation was supposed to have contributed to the striking difference between the hatching rates of the tropical strain in two consecutive winter seasons in Germany [46]. Besides, cold acclimation could be demonstrated for larvae of *Ae. aegypti* exposed to temperatures as low as −6 °C quite recently [62]. Therefore, it is feasible to assume that cold acclimation also occurs in tropical *Ae. albopictus* strains. Consequently, the distinct natural day-night cycle of the temperature, which typically appears in temperate geographical zones, might enhance the tolerance against low temperatures due to the occurrence of cold acclimation on a small scale.

It can be stated that the controls exposed to a constant temperature of 5 °C do not always exhibit a higher hatching rate as the treatments. However, there was no significant influence between the hatching rates of the winter control and the ones of the treatments. There are two possible explanations for this phenomenon. First, the winter controls were not exposed to optimal developmental temperatures like the summer controls kept at 25 °C. Consequently, the hatching rates were lower than in the summer controls, and in several cases even lower than those of the respective treatments. Secondly, the insignificant hatching rates of the winter control and the treatments demonstrate that a minimum temperature as low as − 5 °C is not relevant for the resulting hatching rate. However, this is not the case for the tropical strain for longer exposure periods, which perfectly corresponds to the lower tolerance of this strain against low temperatures.

When considering hatching in *Ae. albopictus*, a variety of other factors than temperature and duration of cold exposure like humidity, the age of the eggs or the hatching medium play a role [44, 63]. The present study primarily concentrates on hatching after egg exposure to low temperature conditions depending on exposure times. Furthermore, the artificial standardisation of freeze-thaw cycles to a constant time period of eight hours caused differences in chilling, varying from 1.25 °C/h in the 5K group to 3.75 °C/h in the 15K group. It cannot be excluded that different degrees of acclimation resulted and had an influence on the hatching results. However, this factor has to be considered as a cofactor in these experiments as it is linked to a certain temperature regime and cannot be regarded or analysed separately. Future experiments could show how important the speed of chilling is for the hatching rate.

When analysing the hatching patterns, the factor ‘temperature’ had the strongest impact of all examined factors, based on its Cramér´s value (CV). According to benchmarks for the CV proposed by Cohen [64], the temperature has a large effect, whereas the scenario has a medium and the other factors have a small effect on the hatching pattern. The occurrence of delayed hatching at temperatures as low as −10 °C could be interpreted as a warning signal that physiological limits of the strain are approaching. Unfortunately, the analysis of such hatching patterns has not yet been addressed in ecological experiments with *Ae. albopictus*. Our results suggest that this aspect, especially the consideration of delayed hatching, might be worthwhile in future examinations.

For a further spread of *Ae. albopictus* in temperate climate zones, the mean January temperature is often quoted as an important feature for its survival and, thus, its ability of overwintering [16, 31]. For modelling climatic suitability of certain areas for overwintering, a mean January temperature of 0 °C is a common threshold. However, Waldock et al. [65] argued for an adaptation of this threshold to −4 °C as this value leads to a better match of mean temperature with demonstrated species occurrence. This is further supported by studies demonstrating overwintering at temperatures lower than 0 °C. The mean January temperature can actually vary between −2 °C for Japan [66], −3 °C for populations in China and South Korea and even −5 °C for those in the USA [1]. However, permanent populations are also found at Chinese locations with a mean January temperature of −5 °C [67]. Our study suggests that the mean temperature at which survival is possible is lower than −2 °C, which would be in agreement with findings linked to an *Ae. albopictus* population established in central Germany [29]. Mean temperatures give a good estimate for the suitability of certain areas on larger scales for mosquito species in general but minimum temperatures are more important on smaller scales as these define the survival with respect to microclimate [65]. The laboratory experiments showed that exposure to −10 °C for longer than 10 days resulted in survival of the subtropical and the temperate strains which could similarly be shown for a temperate strain in the USA [43]. Hence, our findings confirm those of Thomas et al. [42] and emphasise the importance of minimum temperatures, in addition to mean temperatures, for estimating *Ae. albopictus* survival during cold spells. Furthermore, as temperate regions are affected by a daily temperature cycle, the temperature range also plays a role in interpreting the survival potential in winter. However, this factor often remains obscure as most laboratory experiments concentrated on constant temperatures. Our experiments indicate that fluctuating temperatures increase the hatching rate, possibly due to cold acclimation. The average temperature in Germany was 0.6 °C in January 2019 and was only slightly warmer than the long-term trend [68]. Thus, the 10K group reflects the winter conditions in Germany best. It has to be considered, however, that maximum and minimum temperatures may vary considerably in different areas of Germany. For instance, there are relatively warm regions in western Germany that exhibit daily temperature ranges similar to the 5K group regime and relatively cold regions in the eastern part of the country where daily temperature variations resemble the 15K group regime. However, it is not clear, if daily temperature ranges larger than 15K could lead to adverse results. Furthermore, it has to be noted that winter temperatures do not define the presence and absence of *Ae. albopictus* alone. Other climatic factors such as summer temperature and precipitation, but also ecological ones like the availability of breeding sites, have to be taken into account when modeling the potential spread of this species on a regional or even global scale [5, 16, 65]. Despite this, the ability to survive the winter due to its physiological plasticity is one of the main driving factors for the northward spread of *Ae. albopictus* [5].

Correspondingly, another important aspect influencing the spread of *Ae. albopictus* is global warming. Rising mean temperatures which can be observed globally will also lead to an increase of mean temperatures in winter seasons. Thus, the last five years (2015–2019), which were the warmest ever recorded in Germany, were characterised by exceptionally mild winters [69]. However, climatic projections do not only predict that the annual number of frost days will decrease in the future throughout Europe, but also the persistence of extreme cold weather events [70, 71]. Although these cold extremes will be less frequent [71], they will have an influence on the overwintering capability of *Ae. albopictus* in temperate regions. Actually, models considering a variety of factors, in addition to winter temperatures, suggest an increased climatic suitability for *Ae. albopictus* in many regions of Germany due to climate change. In particular, western and southern Germany will be at high risk for future establishment of this mosquito species [30–33]. In light of these studies, it can be assumed that suitable areas for a successful overwintering and thus the distribution range of *Ae. albopictus* will further increase rather than decrease in the future. Since the tiger mosquito is an efficient vector of numerous pathogens, including dengue and chikungunya viruses, its spread and establishment is in the long term assumed to be followed by mosquito-borne disease cases or even outbreaks.

## Conclusions

Our results reveal that the eggs of *Ae. albopictus* are able to survive low temperatures for a much longer exposure period than 24 hours. Low temperature tolerance in the tropical and subtropical strains seems to be much higher as previously indicated [42]. In contrast to field experiments carried out formerly [46], much longer exposure periods could be tested in the laboratory experiments presented here. Therefore, this study could elucidate physiological limits at which hatching is possible as well as critical exposure periods much better than field experiments. Recent models could show that there is a high probability of spread of *Ae. albopictus* especially in western and southern parts of Germany usually characterised by mild winters [30–33]. Our results strongly attest the capability of *Ae. albopictus* to overwinter in regions with winter minimum temperatures of −10 °C, being in agreement with findings in the field [29, 46, 72]. These results together with the indication of short-term tolerance of this species against temperatures as low as −12 °C [42] should be considered in future projections and justify comprehensive mosquito monitoring programmes, also including the public [19].

## Data Availability

The datasets used and/or analysed during the present study are available from the corresponding author on reasonable request.

## Abbreviations

ANOVA: analysis of variance
CV: Cramér’s Value
EDTA: Ethylenediaminetetraacetic acid
HSD: honestly significant different
